# The size of the treatment effect: do patients and proxies agree?

**DOI:** 10.1186/1471-2377-9-12

**Published:** 2009-03-25

**Authors:** Femke AH van der Linden, Jolijn J Kragt, Jeremy C Hobart, Martin Klein, Alan J Thompson, Henk M van der Ploeg, Chris H Polman, Bernard MJ Uitdehaag

**Affiliations:** 1 Department of Neurology, VU University Medical Centre, Amsterdam, The Netherlands; 2 Medical Psychology, VU University Medical Centre, Amsterdam, The Netherlands; 3 Clinical Epidemiology and Biostatistics, VU University Medical Centre, Amsterdam, The Netherlands; 4 Neurological Outcome Measures Unit, Institute of Neurology, London, UK; 5 Peninsula Medical School, Derriford Hospital, Plymouth, UK

## Abstract

**Background:**

This study examined whether MS patients and proxy respondents agreed on change in disease impact, which was induced by treatment. This may be of interest in situations when patients suffer from limitations that interfere with reliable self-assessment, such as cognitive impairment.

**Methods:**

MS patients and proxies completed the Multiple Sclerosis Impact Scale (MSIS-29) before and after intravenous steroid treatment. Analyses focused on patient-proxy agreement between MSIS-29 change scores. Transition ratings were used to measure the patient's judgement of change and whether this change was reflected in the MSIS-29 change of patients and proxies. Receiver operating characteristic (ROC) analyses were also performed to examine the diagnostic properties of the MSIS-29 when completed by patients and proxies.

**Results:**

42 patients and proxy respondents completed the MSIS-29 at baseline and follow-up. Patient-proxy differences between change scores on the physical and psychological MSIS-29 subscale were quite small, although large variability was found. The direction of mean change was in concordance with the transition ratings of the patients. Results of the ROC analyses of the MSIS-29 were similar when completed by patients (physical scale: AUC = 0.79, 95% CI: 0.65 – 0.93 and 0.66, 95% CI: 0.48 – 0.84 for the psychological scale) and proxies (physical scale: 0.80, 95% CI: 0.72 – 0.96 and 0.71, 95% CI: 0.56 – 0.87 for the psychological scale)

**Conclusion:**

Although the results need to be further explored in larger samples, these results do point towards possible use of proxy respondents to assess patient perceived treatment change at the group level.

## Background

The clinical course of Multiple Sclerosis (MS) has a variable pattern and the impact on daily life of patients will increase over time [1]. Treatment of MS is aimed at reducing this impact and to demonstrate this it is essential that disease impact is measured in a reliable and valid way. Due to the limited relation between 'objective' measures like neurological examination and MRI and disease burden as experienced by the patient, these measures are questionable as main outcomes in rehabilitation and therapeutic trials [2]. Recently and in line with this, there is an increasing recognition and use of self-report measurements in clinical settings, in order to capture the patients' perspective. The incorporation of self-report measurements in clinical research also has a downside; there are several patient groups and situations in which the ability to complete a questionnaire may be impaired. Conditions such as cognitive impairment or mood disturbances, which also might play a role during the disease course of MS [3-6], could lead to inaccurate self-report or even loss of information due to missing data. This could result in data which are not representative for the patient population of interest. Exclusion of such patients, a sometimes chosen approach, may cause bias in the assessment of health status. A possible solution for this problem is use of proxy respondents (like the patients' partner) as an alternative source of information [7]. They can provide information on the health status of the patient that otherwise would be inaccurate or even lost. In a previous cross-sectional study we found that patient-proxy agreement was good and proxy respondents might be of value in MS research [8]. However, since factors such as cognitive impairment and mood disturbances are not fixed, the validity of measuring changes over time, which is a crucial requirement for rehabilitation and trials, is especially vulnerable. Proxy respondents of MS patients should therefore also be able to assess change which is induced by treatment. The objective of this study was therefore to examine whether MS patients and proxy respondents agreed on change in disease impact which was induced by treatment.

## Methods

### Study sample

This study was performed at the MS Center of the VU University Medical Center. MS patients, who visited the center in order to receive intravenous steroid treatment for worsening disease symptoms, were approached to participate in the study. The inclusion criteria were that the patient had to have a partner with whom they were living together and this person had to be willing to participate as a proxy respondent for the patient. The medical ethical committee of the VU University Medical Center approved the study protocol and all participants gave informed consent.

### Measures and procedures

Assessment of patients and proxy respondents took place before the start of the intravenous steroid treatment and six to eight weeks after treatment. This follow-up period was chosen, because this would most likely represent the time in which change in disease status could be expected [9]. Both patients and proxy respondents were asked to complete the Multiple Sclerosis Impact Scale (MSIS-29), at the initial visit at the MS Center and again at follow-up when it was posted by mail.

The MSIS-29 measures disease impact of MS on daily life and can be divided into two subscales; a physical scale which consists of 20 items and a psychological scale which consists of 9 items. Scores on the individual items are added and transformed to a 0–100 scale, thereby generating two summary scores of both scales. Higher scores indicated higher impact of MS on daily life [10]. The proxy respondents completed a modified version of the MSIS-29 in which all items were phrased in the third person perspective. The proxy respondents were instructed to assess the patient as the proxy respondent thought the patient would rate himself or herself [11]. They had to complete the MSIS-29 keeping in mind the following question: 'How do you think the patient experiences the impact of MS on his/her life?' The explicit instructions were given that the proxy respondent had to complete the questionnaires independently from the patient.

At follow-up, global ratings of the patient regarding their extent of recovery from their recent deterioration were collected. This was done by using a transition question, which required the patient to compare their current health status to their health status before treatment [12]. In this study, patients were asked: 'In what way, do you believe, is your situation regarding your MS recovered in comparison to before the treatment?' Answer categories included the following items: 'not at all' – 'a little' – 'moderately – 'quite a lot' – 'completely'. For analyses purposes these items were later dichotomized into *not improved *('not at all' – 'a little') and *improved *('moderately' – 'quite a lot' – 'completely'). The Expanded Disability Status Scale (EDSS) and the MS subtype were available for all patients.

### Data analyses

Two different approaches were used to analyse the data. First, we focused on the absolute mean change scores on the MSIS-29 of both patient and proxy respondents and whether they agreed on the direction and the amount of change. Therefore, the overall mean MSIS-29 scores and overall mean change scores (follow-up minus baseline) of the MSIS-29 were calculated for both scales. The mean change scores were standardized by calculating effect sizes (mean change/standard deviation of mean change) [13]. Independent t-tests were used to see whether the mean change scores of patients and proxy respondents differed significantly from each other. Also, the correlation between ratings of patients and proxy respondents was calculated with the intraclass correlation coefficient (ICC), which is the ratio of the variance between subjects (variance of interest) and the total variance [14-16]. For this study the two-way random model for absolute agreement was used [17]. Standards for interpreting ICC values are arbitrary but one can apply the standard reliability criteria of an ICC > 0.70, which is adequate and an ICC > 0.80 is preferred [18].

In the second approach we focused on the transition rating of the patient. The transition rating was regarded as the 'gold standard' and it was examined whether the patient's judgement of change was reflected in the change on the MSIS-29 of both patients and proxy respondents. The patient sample was therefore divided into two groups; the group in which the patients thought they had not improved after treatment and the group in which the patients thought they had improved after treatment. In both groups, mean change scores were calculated for patients and proxy respondents. Again, independent t-tests were used to see whether the differences between these change scores were significant. This time, responsiveness ratios were calculated. Responsiveness ratios relate a clinically relevant change to the variability of the change score in stable patients (mean change in improved group/standard deviation of mean change in not improved group) [19]. ICCs were also calculated. Scatterplots were made to visualize the distribution of individual patient-proxy couples according to the transition ratings and whether the changes on the MSIS-29 were in concordance with the direction of the transition ratings.

The dichotomized transition ratings were also used in receiver operating characteristic (ROC) analyses to see whether the diagnostic properties of the MSIS-29 were similar when completed by patients or proxy respondents. Optimal cut-off points were also determined by means of the ROC curve; the point most upper left in the curve represents the most optimal cut-off point under the assumption that sensitivity to change is equally important to specificity to change [20]. Using this optimal cut-off point sensitivity and specificity values were determined. The sensitivity of the MSIS-29 is the proportion of importantly improved persons according to the transition rating, who are correctly identified by the MSIS-29 as importantly improved [21]. The specificity of the MSIS-29 is the proportion of the persons who are not improved according to the transition rating, who are correctly identified as not improved by the MSIS-29 [21]. In addition, positive predictive values (PPV) and negative predictive values (NPV) were calculated. The PPV is the proportion of patients identified as improved by the MSIS-29 who are also improved according to the transition rating. The negative predictive value is the proportion of patients below the cut-off of the MSIS-29 who were not improved according to the transition score. Furthermore, the area under the ROC curve (AUC) was calculated, which represents the probability that the MSIS-29 correctly classified patients as improved or unimproved [22]. The larger the value of the AUC, the better the ability of the MSIS-29 to distinguish between patients who did and did not experience an important change.

## Results

### Sample characteristics

From October 2004 until October 2006, 55 patient-proxy couples initially agreed to participate in the study. Missing data was mainly caused by the fact that the MSIS-29 was not returned at follow-up (nine patient-proxy couples), despite telephone reminders. One patient and one proxy respondent had more than 50% missing items on both scales of the MSIS-29 and were therefore left out of the analyses [23]; one patient withdrew from the study due to worsening disease status and one patient-proxy couple did not complete the MSIS-29 independently from each other. Table 1 shows the characteristics of the remaining 42 patients and proxy respondents. Seven of these 42 patients already participated in an earlier published study on longitudinal proxy measurements [24].

**Table 1:** Characteristics of patients and proxy respondents

	**Patients**	**Proxy respondents**
Total (n)	42	42
Female (n)	22	22
Age (years)*	45.7 (9.7)	47.7 (10.3)
Years since MS onset*	10.5 (6.1)	

Type of MS (n)		
Relapsing remitting	26	
Secondary progressive	10	
Primary progressive	6	
		
EDSS baseline **	4.8 (2.5 – 8.0)	
EDSS after treatment**	4.3 (1.0 – 8.0)	

*Values are mean (SD)

** Values are median (range)

EDSS; Expanded Disability Status Scale

Table 2 displays the results of the comparison between the absolute mean change scores on the MSIS-29 of patients and proxy respondents. Both patients and proxy respondents showed negative mean change scores on the physical scale. This indicates a lower score on the MSIS-29 physical scale after treatment, which subsequently pointed towards an overall decrease in physical disease impact after treatment. Patients also showed a decrease on the psychological scale (-3.7 ± 15.2) but the proxy respondents remained essentially unchanged (-0.1 ± 20.2). All mean values were accompanied by large standard deviations, indicating large variability on in the individual patient-proxy level.

**Table 2:** Overall mean MSIS-29 scores at baseline and after treatment

	**Patients** **Mean ± SD** **(n = 42)**	**Proxy respondents** **Mean ± SD** **(n = 42)**
MSIS-29 physical scale baseline	48.8 ± 18.4	48.2 ± 18.0
MSIS-29 physical scale after treatment	42.0 ± 20.3	44.6 ± 20.8
Change on MSIS-29 physical scale^a^	-6.8 ± 16.3	-3.6 ± 16.2
Effect size	0.4	0.2

Difference between change scores^b^:	-3.2 ± 14.8 (p = 0.367)	
95% CI of the difference	(-10.3 – 3.8)	
ICC	0.58	

MSIS-29 psychological scale baseline	31.4 ± 19.6	34.7 ± 20.4
MSIS-29 psychological scale after treatment	27.7 ± 17.8	34.6 ± 21.1
Change on MSIS-29 psychological scale^a^	-3.7 ± 15.2	-0.1 ± 20.2
Effect size	0.2	0.0

Difference between change scores^b^:	-3.6 ± 20.5 (p = 0.353)	
95% CI of the difference	(-11.4 – 4.1)	
ICC	0.34	

^a ^mean MSIS-29 scores after treatment minus mean MSIS-29 scores at baseline; negative values indicate improvement

^b ^Patient mean change score minus proxy mean change score

CI = Confidence Interval ICC = Intraclass correlation coefficient

Patients indicated a larger mean change on both scales in disease impact in comparison to the proxy respondents, which was reflected in the larger effect sizes. However, independent t-test showed that these differences on both scales were not significant (p = 0.05). ICCs were 0.58 for the physical scale and 0.34 for the psychological scale, indicating moderate to low agreement.

Table 3 shows the results of the analyses according to the transition ratings. From the 42 patients, 25 patients indicated that they had not improved after treatment and 17 patients indicated that they had improved after treatment.

**Table 3:** Mean MSIS-29 scores and change scores for patients and proxies at baseline and after treatment according to the transition ratings

	Not improved^a ^(n = 25)		Improved^b ^(n = 17)	
MSIS-29	Patients	Proxy respondents	Patients	Proxy respondents
Physical scale baseline	51.3 ± 16.5	51.0 ± 15.0	45.2 ± 20.9	44.2 ± 21.4

Physical scale after treatment	51.1 ± 16.8	55.0 ± 15.0	28.8 ± 17.7	29.5 ± 19.0
Change on MSIS-29 physical scale^c^	-0.2 ± 12.8	4.0 ± 13.5	-16.4 ± 16.4	-14.7 ± 13.3

Responsiveness ratio	1.29		1.09	

Difference between change scores^d^:	-4.2 ± 14.7 (p = 0.948)		-1.8 ± 15.1 (p = 0.733)	
95% CI of the difference	(-11.7 – 3.3)		(-12.2 – 8.6)	
ICC	0.36		0.50	

Psychological scale baseline	30.7 ± 19.1	33.2 ± 18.3	32.5 ± 20.9	36.9 ± 23.5
Psychological scale after treatment	30.7 ± 17.7	40.1 ± 22.3	23.4 ± 17.6	26.6 ± 16.8
Change on MSIS-29 psychological scale^c^	0.0 ± 14.3	6.9 ± 19.9	-9.1 ± 15.3	-10.3 ± 16.3

Responsiveness ratio	0.64		0.52	

Difference between change scores^d^:	-6.9 ± 18.3 (p = 0.544)		1.1 ± 23.1 (p = 0.834)	
95% CI of the difference	(-16.7 – 3.0)		(-8.9 – 12.2)	
ICC	0.42		0.0	

^a ^combines the transition ratings 'not at all' – 'a little'

^b ^combines the transition ratings 'moderately' – 'quite a lot' – 'completely'

^c ^mean MSIS-29 scores after treatment minus mean MSIS-29 scores at baseline (negative values indicate improvement)

^d ^patient mean change score minus proxy mean change score

CI = Confidence Interval

ICC = Intraclass correlation coefficient

The patients who considered themselves not improved showed a very small decrease at group level for the physical scale (-0.2 ± 12.8) and no mean change at group level on the psychological scale (0.0 ± 14.3). Yet, standard deviations of the change scores were large.

Proxy respondents, on the other hand, indicated a small increase in disease impact on both scales when the patients indicated they did not improve: 4.0 ± 13.5 on the physical scale and 6.9 ± 18.3 on the psychological scale. Again, the variability was large. The differences between patients and proxy respondents were not significant (p = 0.05). ICCs between patient and proxy scores were low for both scales; 0.36 for the physical scale, 0.42 for the psychological scale. The responsiveness ratio for the physical scale was 1.29 and 0.64 for the psychological scale.

In the improved group, both patients and proxy respondents showed large negative mean change scores, indicating less disease impact on the MSIS-29 after treatment. Differences between the changes scores for patients and proxy respondents appeared to be small, but standard deviations were large. Hence, the differences were not significant (p = 0.05). The ICC for the physical scale was moderate (0.50) and extremely low for the psychological scale (0.0). The responsiveness ratios were lower in the improved group: 1.09 for the physical scale and 0.52 for the psychological scale.

**Figure 1:**
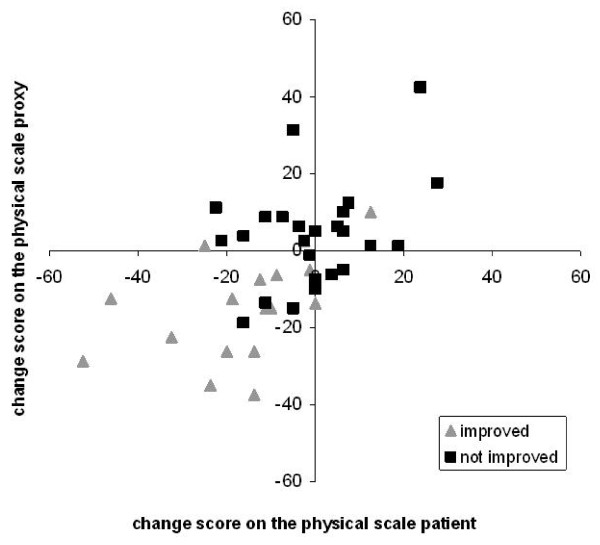
**Individual change scores for patients and proxy respondents on the physical scale**.

**Figure 2:**
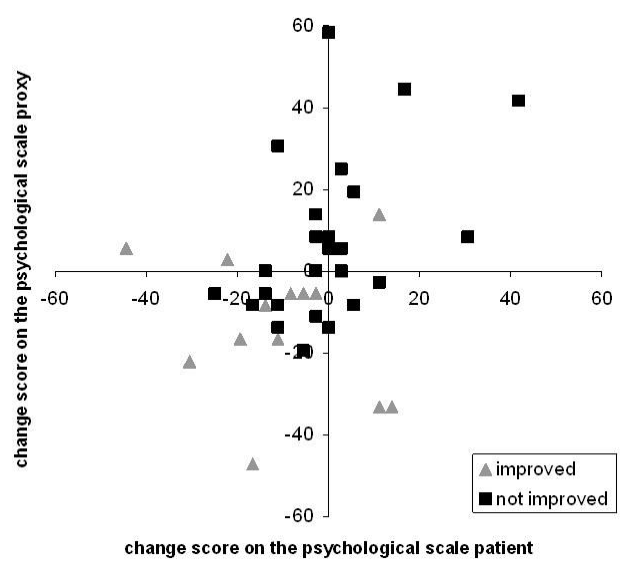
**Individual change scores for patients and proxy respondents on the psychological scale**.

Figure 1 and 2 are scatterplots of the individual change scores of the patient-proxy couples in the improved group and the group who did not improve as defined by the patient's reported transition question.

In figure 1, the individual change scores on the physical scale of the improved group were predominantly (14 patient-proxy couples; 71%) located in the lower, left quadrant which corresponded with negative change scores on the MSIS-29. The individual change scores on the physical scale of the not improved group were more scattered among the four quadrants.

In figure 2, the individual change scores on the psychological scale of the improved group were also mostly located in the lower left quadrant (10 patient-proxy couples; 58%). The individual change scores for the psychological scale of the not improved group were also scattered among the four quadrants.

Table 4 displays the results of the ROC analyses for patients and proxy respondents. The most optimal cut-off point for change on the physical scale, when completed by patients, was -8.13. This value corresponded with a sensitivity of 76% and a specificity of 76%. In case of a change score of -8.13 or more on the physical scale of the MSIS-29, 82% of the patients actually had changed (PPV). In case of a change score below this point, 68% of the patients actually had not changed (NPV). The cut-off point for proxy respondents on the physical scale was -6.88, which corresponded with a sensitivity of 80% and a specificity of 71%. The positive predictive value in this case was 80% and the negative predictive value was 71%.

**Table 4:** Results of ROC analyses

	**MSIS-29**	**Cut-off point**	**Sensitivity**	**Specificity**	**PPV**	**NPV**
Patients	Physical scale	-8.13	76%	76%	82%	68%
	
	Psychological scale	-5.56	72%	65%	74%	65%

Proxy respondents	Physical scale	-6.88	80%	71%	80%	71%
	
	Psychological scale	-4.17	64%	71%	76%	57%

PPV, positive predictive value; NPV, negative predictive value

The most optimal cut-off point for the psychological scale, when completed by patients was -5.56, which corresponded with a sensitivity of 72% and a specificity of 65%. In case of a change score of -5.56 or more, 74% of the patients had changed. In case of a change score below this point, 65% of the patients had not changed. The cut-off point for proxy respondents, when they complete the psychological scale, was -4.17. This cut-off point corresponded with a sensitivity of 64% and a specificity of 71%. The positive predictive value in this case was 76% and the negative predictive value was 57%.

**Figure 3:**
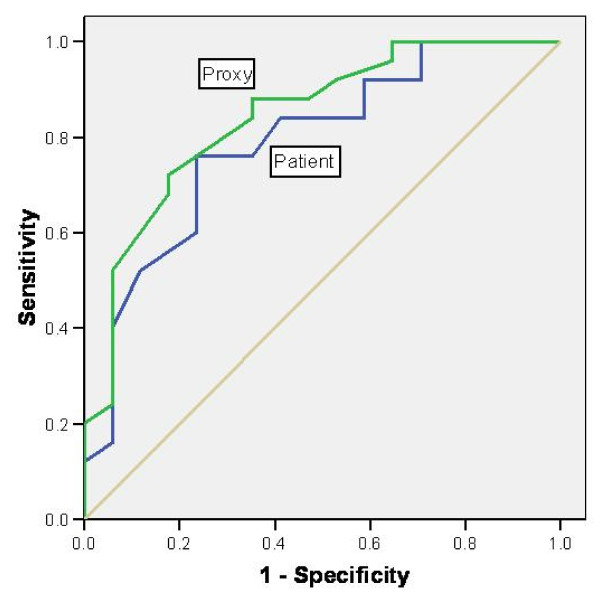
**ROC curves for the MSIS-29 physical scale**.

**Figure 4:**
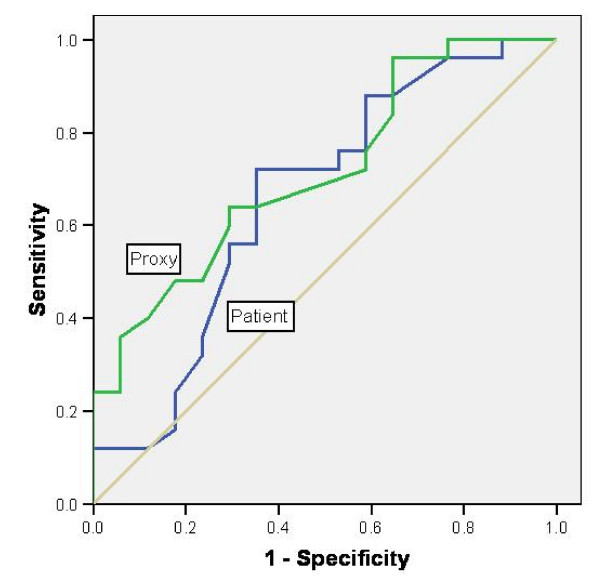
**ROC curves for the MSIS-29 psychological scale**.

ROC curves are presented in figure 3 and 4. The AUC values for the patients were 0.79 (95% CI: 0.65 – 0.93) for the physical scale and 0.66 (95% CI: 0.48 – 0.84) for the psychological scale. The same AUC values for the proxy respondents were slightly larger at 0.80 (95% CI: 0.72 – 0.96) and 0.71 (95% CI: 0.56 – 0.87).

## Discussion

The objective of this study was to examine whether proxy respondents agreed with MS patients on treatment induced change in disease impact. Ratings of treatment induced change by proxy respondents may be useful for patients with cognitive impairment or mood disorders or other problems that would otherwise exclude them from the study.

Data were analysed using two different approaches: the first focussed on the comparison of the absolute mean change scores on the MSIS-29 between patients and proxy respondents; the second focussed on the transition ratings as an external criterion to evaluate the change on the MSIS-29. The latter approach was used to examine whether repeated MSIS-29 assessment was capable of capturing changes that were indicated by the patient themselves.

The first approach showed small differences between the change scores indicating that there was acceptable agreement between patients and proxy respondents on change in disease impact after intravenous steroid treatment. However, the variability on individual patient-proxy level was large and ICCs were poor to moderate, indicating a low level of agreement. However, since an ICC is based on the variance of the sample, a lack of variance in change scores could also have caused the low ICCs, rather than lack of patient-proxy agreement [15]. Possibly, when patients stayed stable, differences between the two measurements could have been caused by measurement error or random error, which could have lowered the calculated ICCs.

Effect sizes suggested that the apparent treatment effect was greater for patients in comparison to the proxy respondents, but after division into the improved and not improved group this difference diminished.

The second approach illustrated that less patients felt that they had improved (40%) than not improved and patient-proxy agreement in the improved group was better than in the not improved group. There was a clear difference in the amount of change between the not improved and improved group, on both scales. This finding was underlined by the scatterplots and the responsiveness ratios; the individual change scores for the patient-proxy couples in the improved group were mainly located in the lower left quadrant, which corresponded with less disease impact after treatment. The individual changes in the not improved group were more scattered around the quadrants. Responsiveness ratios were also larger in the improved group when compared to the not improved group. The ROC analyses showed similar sensitivity en specificity values for both patients and proxy respondents. Also, the values for the PPV and the NPV were similar and the AUC was even slightly larger for proxy respondents on both scales. These results indicated that the diagnostic characteristics of the MSIS-29 were similar when completed by patients and proxy respondents. It is interesting to note that a recently published study by Costelloe et al. obtained similar values for the physical scale of the MSIS-29 after performing an ROC analysis [25].

Several limitations should be taken into account when interpreting these results. First of all, the attrition rate was high, mainly caused by the fact that the MSIS-29 was not returned at follow-up. This could have caused selection bias. The final results in this study are based on a small sample size of 42 patients. Therefore, current findings should be interpreted with caution. Larger samples will provide the opportunity to create more subgroups based on, for example, disability of the patient. Also, given the small sample sizes, there might not have been enough power to detect a true difference in scores in the subgroups (not improved, improved). There was no data collected on cognitive status or mood disturbances in this sample and we can therefore not make any assumptions on whether these factors might have influenced the results. There has been criticism on the use of transition ratings which could introduce methodological problems [26]. There is evidence that patients have difficulty with recalling prior health status and their judgement is therefore based on their present state, rather than on change in health status [27]. It should be noted that the answer categories of the transition question did not have the option to indicate that the patient felt worse if this should be the case. This could give more insight into the patients who indicated that they did not change at all.

## Conclusion

This is the first study to explore patient-proxy agreement in an intervention setting in MS research. Despite its limitations and although the results need to be further explored in larger samples, these results point towards possible use of proxy respondents to assess patient perceived treatment change at the group level.

## Competing interests

The authors declare that they have no competing interests.

## Authors' contributions

FAHL participated in the data collection and coordination, performed the statistical analysis together with BMJU, and drafted the manuscript. JJK contributed to the data collection and drafting of the manuscript. BMJU conceived of the study concept and participated in its design and coordination and drafting of the manuscript together with JCH, MK, AJT, HMP, and CHP. All authors read and approved the final manuscript.

## Pre-publication history

The pre-publication history for this paper can be accessed here:


